# The complexity of Rab5 to Rab7 transition guarantees specificity of pathogen subversion mechanisms

**DOI:** 10.3389/fcimb.2014.00180

**Published:** 2014-12-22

**Authors:** Giovanna Mottola

**Affiliations:** ^1^UMR MD2, Faculté de Médecine NORD, Aix Marseille University and Institute of Research in Biology of the French ArmyMarseille, France; ^2^Laboratory of Biochemistry, La Timone University Hospital, Assistance Publique Hôpitaux de MarseilleMarseille, France

**Keywords:** bacteria, pathogens, Rab5, Rab7, phagosome

Non-pathogenic bacteria are commonly eliminated by the host. Professional phagocytic cells of the immune system, such as macrophages and dendritic cells, recognize and by phagocytosis internalize microbes in specialized endocytic compartments called phagosomes. By fusing with endosomes, phagosomes mature, changing from an early to late state, and early and late endosomes differ in their external and internal biochemical composition. Then, fusion of late phagosomes with lysosomes leads to the formation of an acidic and degradative compartment, the phagolysosome, where bacteria are ultimately eliminated (Fairn and Grinstein, 2012). Bacterial pathogens have evolved several mechanisms to subvert the process of phagosome maturation and to survive and replicate in an intracellular niche that is protected from the immune response. Remarkably, distinct bacterial pathogens can be localized in similar endocytic compartments, suggesting they have a role in the control of the same endocytic steps. The biological machineries controlling endocytosis involve a variety of regulatory events in each step of intracellular membrane trafficking. Here, I would like to summarize and comment on all the discoveries on bacterial pathogens that control the localization or function of the small GTPases Rab5 and Rab7, and therefore modify the maturation from early to late phagosomes, because I believe such a transition is the best way to highlight how bacterial pathogens exploit the complexity of membrane trafficking to establish specific subversion mechanisms.

Phagosome maturation is highly dependent on the endocytic pathway and requires several regulators of this pathway. Rab proteins make up a large family of small GTPases that specifically control various steps in the endosomal and phagosomal transport process (Gutierrez, 2013; Sherwood and Roy, 2013). Each Rab protein is associated with an organelle and a specific step in intracellular trafficking and controls several factors, such as protein and lipid composition of an organelle membrane, fusion between distinct compartments, vesicle motility along microtubules, and interaction with the cytoskeleton. Each compartment has a specific and precise set of Rab proteins, which confers organelle identity. The complexity of Rab protein function is reflected in their life cycle. All Rabs alternate between an active (GTP-bound) state and an inactive (GDP-bound) state. This molecular switch is strictly regulated; one or more guanine nucleotide exchange factors (GEFs) catalyze the release of GDP in exchange for GTP. In the GTP-bound form, Rabs are targeted to their specific organelle membrane by prenylation and recruit a large number of downstream effectors. In the GDP-bound form, they associate with a soluble factor, the guanine dissociation inhibitor (GDI), which stabilizes the inactive species in the cytosol and precludes access to the GEFs. The hydrolysis of GTP to GDP by GTPase-activating proteins (GAPs) terminates the activity of the Rab protein until another activation cycle is initiated.

Rab5 and Rab7 are the best-characterized Rab proteins in the endocytic process and, especially by analogy, in phagosome maturation (Fairn and Grinstein, 2012). Rab5 is mainly associated with early endosomes and early phagosomes and controls the identity and functionality of these compartments. Rab7 defines late endosomes and late phagosomes, and it has been implicated in the transport through these compartments. Therefore, when internalized, bacteria are first localized in Rab5-positive phagosomes and then in Rab7-positive phagosomes. There is much debate about the mechanisms whereby an early Rab5-positive endosome or a phagosome becomes a late Rab7-positive endosome or phagosome and whereby Rab5 is consecutively replaced by Rab7 and much effort has been given to understanding the regulation of Rab5 and Rab7 function. The picture so far shows Rabs as complex, highly regulated molecular machineries. A large number of effector proteins interacting with each Rab and of the GEFs and GAPs that regulate their function have been described. In the case of Rab5, for example, more than 60 effectors have been found and several remain to be characterized, and at least 4 GEFs of Rab5 have been characterized so far (Horiuchi et al., 1997; Christoforidis and Zerial, 2000; Kajiho et al., 2003; Otomo et al., 2003; Olchowik and Miaczyńska, 2009; Balaji et al., 2012). These discoveries led to a more detailed investigation of the compartment where pathogens reside, which is not simply Rab5- or Rab7-positive, and to a larger comprehension of the molecular mechanisms that bacterial pathogens have evolved. In fact host activities and molecules that pathogens modify the Rab5 to Rab7 transition are different and specific for each pathogen (Figure 1). For example, *Mycobacterium tuberculosis* and *Listeria monocytogenes* have been localized in modified Rab5-positive endocytic compartment. Nonetheless, at molecular level they distinctly affect the Rab5 machinery. *M. tuberculosis*, responsible for the human disease tuberculosis, enters alveolar macrophages and modifies the formation of phosphatidylinositol 3-phosphate [PI(3)P] at the early phagosome membranes (Fratti et al., 2001). The mannose-capped lipoarabinomannan (man-LAM) in the bacterial membrane is released into the phagosomal membrane and inactivates Vps34, the PI(3)P kinase that regenerates PI(3)P (Fratti et al., 2003). At the same time, an *M. tuberculosis* lipid phosphatase, SapM, consumes PI(3)P and arrests phagosome maturation (Saikolappan et al., 2012; Puri et al., 2013). PI(3)P is specifically enriched in early endosome/phagosome membranes and stabilizes Rab5 and all its effectors. Its absence in *M. tuberculosis* infection interferes with the recruitment of the Rab5 effectors and therefore with phagosome maturation to the Rab7 state (Purdy et al., 2005). Interestingly, the *M. tuberculosis*-containing phagosome is also enriched for Rab22a, which inhibits Rab7 acquisition and arrests phagosomal maturation (Roberts et al., 2006).

**Figure 1:**
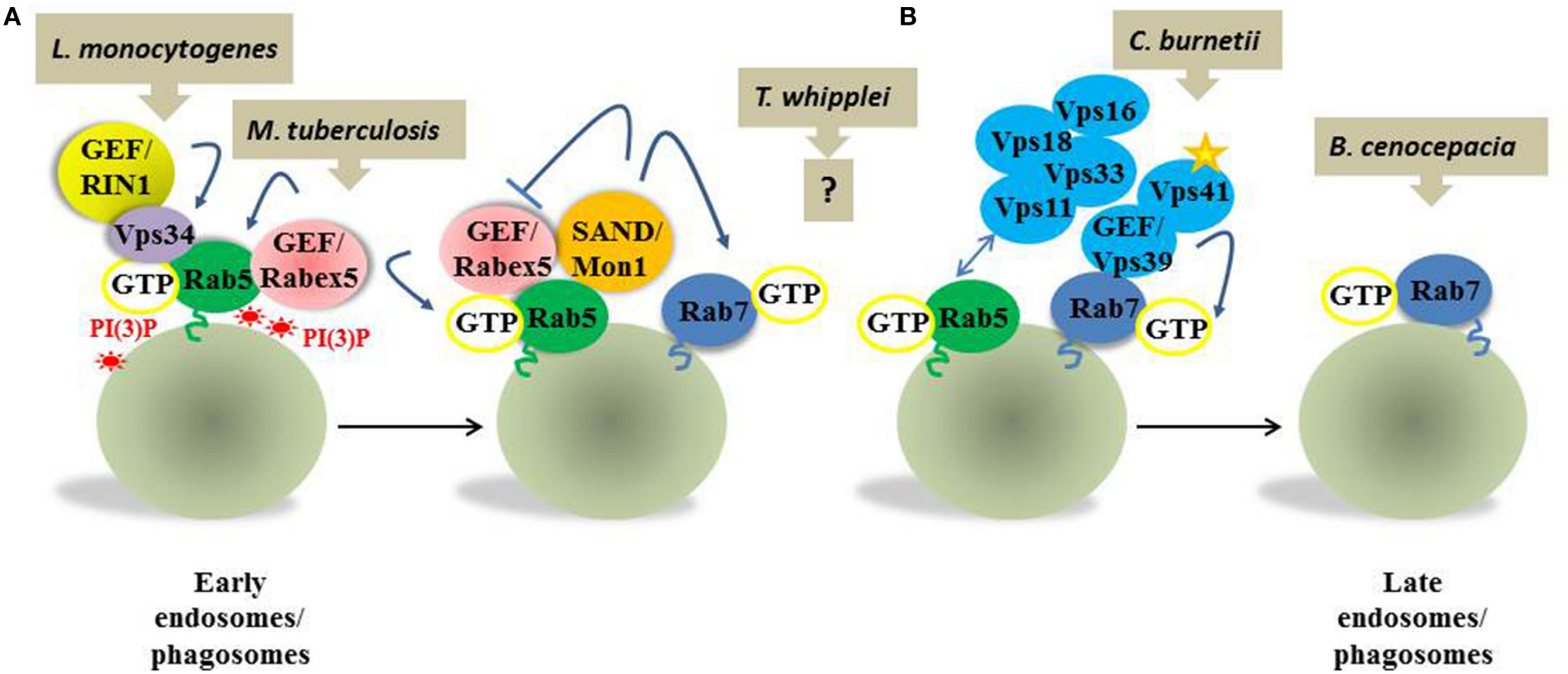
**Simplified view of the molecular mechanisms involved in Rab5 to Rab7 transition exploited by bacterial pathogens**. Rab5 in its active form recruits on the early compartment a GEF Rabex5, which stabilizes Rab5 recruitments to the membrane, and Vps34, the PI(3)P kinase that regenerates PI(3)P. Rab5 activity at the early endosomes/phagosomes is also regulated by GEF RIN1. Two events take place on the early endosomes, which implicate simultaneous recruitment of Rab7 and maturation toward the late endosomes/phagosomes. **(A)** SAND1/Mon1 binds Rabex5 and displaces it from early endosome, inactivating the Rab5 recruitment loop. Additionally, SAND1/Mon1 interacts with a Rab7 GEF, the Vps39 subunit of the HOPS complex (blue). **(B)** The HOPS complex Vps11 subunit interacts with Rab5-GTP, probably stabilizing the Rab5-Rab7 transition. Interestingly p38α-MAPK dependent phosphorylation of the HOPS complex Vps41 subunit also seems important for Rab7 recruitment. Upon Rab7 recruitment and activation, Rab5 is released and early endosomes/phagosomes mature in late endosomes/phagosomes. For each of the described steps a distinct subversion mechanism has been evolved by bacterial pathogens. *L. monocytogenes* engages RIN1 to promote accumulation in a Rab5-positive compartment. *M. tuberculosis* inactivates Vps34 and consumes PI(3)P, interfering with Rab5 recruitment. *T. whipplei* blocks the transition in a Rab5- and Rab7 positive state by an unknown mechanism. *C. burnetii* interferes with Vps41 phosphorylation and Rab7 recruitment. *B. cenocepacia* affects Rab7 activation on the membranes.

A GEF Rabex5-Rabaptin5 complex is recruited by the active GTP-bound form of Rab5 and through a positive feedback loop regulates Rab5 recruitment to the early endosomes (Lippe et al., 2001). Recent evidence suggests that Rab5 activation is also regulated by RIN1, a RAS effector and a Rab5-GEF (Jiwani et al., 2012; Balaji et al., 2014). *Listeria monocytogenes* is a gram-positive food-borne pathogen that causes severe infection with symptoms ranging from gastroenteritis to bacterial meningitis and has a mortality rate of about 30% (Chen et al., 2013). *L. monocytogenes* invades intestinal epithelial cells and survives in a Rab5-positive phagosome until it is prepared to lyse the phagosomal membrane and escape into the cytosol (Farber and Peterkin, 1991). For this purpose, *L. monocytogenes* acts at the level of Rab5 localization and functions in two steps. First, its attachment to the host cell triggers activation of RIN1, which activates Rab5 for efficient internalization by receptor-mediated phagocytosis and transport to early phagosomes (Jiwani et al., 2012; Balaji et al., 2014). Bacteria then need to block Rab5 activity to avoid maturation of early phagosomes into late phagosomes. Thus, the *L. monocytogenes* glyceraldehyde-3-phosphate dehydrogenase (GAPDH) protein ADP-ribosylates Rab5, rendering this GTPase unresponsive to activation by GEFs, and thereby blocks maturation into Rab7-positive phagosomes (Prada-Delgado et al., 2005).

*Tropheryma whipplei* is a nice example of blockade of endocytic trafficking in an intermediate state of the Rab5 and Rab7 transition. This pathogen is responsible for a multi-systemic infection called Whipple's disease, which is fatal without antibiotic treatment (Schneider et al., 2008). *T. whipplei* resides and replicates in both macrophages and non-microbicidal cells in a phagosome that does not become a phagolysosome (Ghigo et al., 2002, 2010). Recently, the purification and characterization of the intracellular compartment where *T. whipplei* localizes revealed that *T. whipplei*-containing compartments are the first example of Rab5- and Rab7-positive phagosomes containing bacteria (Mottola et al., 2014). How is the pathogen establishing this intermediate state? Actually, the transition from a Rab5-positive to Rab7-positive endosome requires both Rabs, and Rab7 is already present on early endosomes together with Rab5 (Poteryaev et al., 2007). Such transition involves two multimeric complexes. The SAND1/Mon1 and ccz1 complex binds Rabex5 and displaces it from the early endosome, inactivating the Rab5 recruitment loop (Figure 1 and Poteryaev et al., 2007). At the same time, the SAND1/Mon1–Ccz1 complex is also a Rab7 GEF (Nordmann et al., 2010; Cabrera et al., 2014). Intriguingly, Rab5 and Rab7 both bind to the hexameric tethering complex HOPS (“homotypic fusion and protein sorting”). Rab5 binds to subunit Vps11 (Rink et al., 2005). Rab7 interacts with subunits Vps39 and Vps41, but SAND1/Mon1 also interacts with Vps39, which in yeast is a Rab7 GEF (Peralta et al., 2010; Plemel et al., 2011). By a hitherto undescribed mechanism *T. whipplei* therefore might affect either the function of the SAND1/mon1-ccz1 complex or of the HOPS complex and block endosomes at an intermediate Rab5- and Rab7-positive state. Further investigation of the mechanism responsible for the presence of both Rab5 and Rab7 on *T. whipplei* phagosomes will help us better understand both the *T. whipplei* infectious process and the regulatory mechanism of the Rab5-to-Rab7 switch.

The investigation of *Coxiella burnetii* subversion mechanisms has also revealed insights on pathogen specificity. *C. burnetii*, the causative agent of the zoonosis Q fever, is also responsible for lethal endocarditis (Raoult et al., 2005). In macrophages, virulent *C. burnetii* bacteria reside and replicate in compartments known as “phagolysosome-like vacuoles” that have properties of both late endosomes and lysosomes. These compartments do not harbor lysosomal enzymes or Rab7, but they have acidic properties and are positive for lysosomal-associated membrane protein-1 (LAMP-1) (Ghigo et al., 2009, 2012). Recently, by using an avirulent form of this pathogen, Barry A.O. et al. discovered that p38α-MAPK-dependent phosphorylation of HOPS complex Vps41 subunit is crucial for Rab7 recruitment to endosomal membranes (Barry et al., 2012). Remarkably, the lipopolysaccharide of virulent *C. burnetii* (LPS), a bacterial outer membrane component, is responsible for this subversion mechanism (Barry et al., 2012). Indeed, it interferes with the activation of p38α-MAPK and therefore with Vps41 phosphorylation. Consequently, *C. burnetii-*containing phagosomes become positive for Rab5, lose Rab5, but do not recruit Rab7.

*Burkholderia cenocepacia* has evolved a distinct mechanism to interfere with early to late phagosome transition. *B. cenocepacia* is an opportunistic pathogen that infects patients with cystic fibrosis (Drevinek and Mahenthiralingam, 2010). It can survive within macrophages because it arrests the fusion of phagosomes with lysosomes by acting at the level of Rab7 function (Lamothe et al., 2007; Lamothe and Valvano, 2008). Vacuoles containing *B. cenocepacia* transiently recruit Rab5 and synthesize PI(3)P. Vacuoles can also acquire the late phagosomal markers CD63 and Rab7, but activation of Rab7 is impaired by the bacteria (Huynh et al., 2010). Findings have indicated that the type III secretion system is not necessary for maturation arrest (Lamothe et al., 2007), and *B. cenocepacia* also expresses type IV and type VI secretion mechanisms (Aubert et al., 2008; Sajjan et al., 2008), but the identities of the secreted effectors and their mode of action on Rab7 remain unclear.

In conclusion, I have described five distinct pathogens that distinctly exploit the complexity of Rab5 and Rab7 regulation in order to survive and replicate in the host environment. Why is that important? First, this example highlights the coevolution of the two systems. Mammalian cells have evolved a complicated regulation of Rab5 and Rab7 transition in order to guarantee redundancy and therefore “resistance” to any possible dangerous genetic or acquired alteration. Also, pathogens more specifically explore transport steps to establish their own pathogen-specific subversion mechanism. Indeed, such specificity guarantees longer survival and evolution before the host immune system becomes able to find and positively select an adequate immune response. Moreover, this example underlines the strong need for multidisciplinary approaches in the study of infectious diseases. To understand pathogen behavior, membrane trafficking in these pathological contests must be investigated biologically and biochemically. This will be determinant in the development of specific prognostic, diagnostic, and therapeutic tools against an infectious pathology.

## Conflict of interest statement

The author declares that the research was conducted in the absence of any commercial or financial relationships that could be construed as a potential conflict of interest.
